# No Longer Confidential: Estimating the Confidence of Individual Regression Predictions

**DOI:** 10.1371/journal.pone.0048723

**Published:** 2012-11-15

**Authors:** Sebastian Briesemeister, Jörg Rahnenführer, Oliver Kohlbacher

**Affiliations:** 1 Applied Bioinformatics Group, Center for Bioinformatics/Dept. of Computer Science, University of Tübingen, Germany; 2 Department of Statistics, TU Dortmund, Dortmund, Germany; Memorial Sloan Kettering Cancer Center, United States of America

## Abstract

Quantitative predictions in computational life sciences are often based on regression models. The advent of machine learning has led to highly accurate regression models that have gained widespread acceptance. While there are statistical methods available to estimate the global performance of regression models on a test or training dataset, it is often not clear how well this performance transfers to other datasets or how reliable an individual prediction is–a fact that often reduces a user’s trust into a computational method. In analogy to the concept of an experimental error, we sketch how estimators for individual prediction errors can be used to provide confidence intervals for individual predictions. Two novel statistical methods, named CONFINE and CONFIVE, can estimate the reliability of an individual prediction based on the local properties of nearby training data. The methods can be applied equally to linear and non-linear regression methods with very little computational overhead. We compare our confidence estimators with other existing confidence and applicability domain estimators on two biologically relevant problems (MHC–peptide binding prediction and quantitative structure-activity relationship (QSAR)). Our results suggest that the proposed confidence estimators perform comparable to or better than previously proposed estimation methods. Given a sufficient amount of training data, the estimators exhibit error estimates of high quality. In addition, we observed that the quality of estimated confidence intervals is predictable. We discuss how confidence estimation is influenced by noise, the number of features, and the dataset size. Estimating the confidence in individual prediction in terms of error intervals represents an important step from plain, non-informative predictions towards transparent and interpretable predictions that will help to improve the acceptance of computational methods in the biological community.

## Introduction

Computational methods, in particular regression methods, are usually praised as a potential replacement of wet lab experiments. Due to their ability to learn patterns and coherences from empirical data they can provide reasonably accurate predictions in a very time-efficient manner. Unfortunately, the credibility of computational models in the biological community is still rather low. One major reason is their “black box” character: Biologists are often left with plain prediction values without any additional error information. Since biologists are not made aware of the fact that predictions can be prone to errors, they are forced to use the regression model as a “black box”, leaving them disappointed in case of less accurate prediction.

In the experimental sciences, the concept of a measurement and its associated error is a cornerstone in understanding the reliability of a data point. Determining these errors is well established, be it through experimental replicates or by considering uncertainty in the input variables. Not specifying the error of an experimental measurement is thus rightly considered a violation of good scientific practice.

Statistical measures to capture the prediction error of computational methods are not a direct replacement for the measurement error. In most cases, it is not even clear what the reliability of a prediction method means. Specifying the correlation coefficient for a training dataset is not sufficient to really give the user an idea of the error of an individual prediction. A further complication is the fact that regression methods are often trained on rather limited datasets. While they maintain good performance on closely related datasets, the error may increase drastically when applied to data points far from the training set. Most of this is usually totally opaque for the user of machine learning methods and hardly ever reported in the popular web servers offering predictions in bioinformatics.

To overcome these problems, confidence estimation, which determines the reliability of individual predictions, is desirable. In cases where highly accurate predictions are required, e.g. for choosing candidates for expensive experiments, confidence intervals would be especially invaluable to biologists.

In classification, the confidence of individual predictions have sometimes already been estimated. Intuitive estimation approaches use the uncertainty between classes, expressed by the posterior probability [1] or the distance to a separating hyperplane [2], to assess the different nature of individual predictions. In contrast, confidence estimators for regression have to utilize properties of the training data or characteristics of the machine learning model [3].

In the area of quantitative structure-activity relationships (QSAR), where regression methods are applied to predict the biological activity of small molecules, the concept of confidence estimation was introduced through so-called applicability domains [4]. The AD defines the input space on which the model is expected to give reliable predictions [5]. However, AD estimators were designed to detect possible extrapolation errors but not to measure the error of instances within the AD. Consequently, some estimators cannot express the confidence in a prediction in a quantitative manner. Although some estimators can provide quantitative scores, it is usually difficult to relate a score to an actual error. Despite some efforts in AD estimation, confidence estimators for regression models have not been applied extensively in the context of computational biology.

It can be distinguished between methods that utilize certain properties of a regression model, e.g. the predictive variance of a Gaussian process, and methods that are independent from a particular regression model. Here, we concentrate on the latter, since model-independent confidence estimators are more universal.

In this work, we introduce a novel concept to confidence estimation. In analogy to experimental measurements, we associate each individual regression prediction with an estimate of its error. We propose two novel confidence estimators, CONFINE and CONFIVE, which return confidence intervals with only a small computational overhead. These intervals contain the real value with a certain probability, while being very small for confident predictions and fairly broad if the prediction is likely to be erroneous. Hence, in contrast to other estimation approaches that only return arbitrary scores, their error estimates are very intuitive and easy to interpret.

CONFINE and CONFIVE estimate the confidence of a prediction by inspecting local properties of the input space. CONFINE determines the error rate of the nearest neighbors of a test instance in the training data. CONFIVE examines the variance in the surrounding local environment and assumes that large variances result in higher error rates. Since both estimators are strictly model-independent, they can be applied with any linear and non-linear regression algorithm.

After presenting related work in this area, we introduce the methods underlying CONFINE and CONFIVE. We discuss their applicability by analyzing the influence of noise, the number of features, and the dataset size on the quality of the estimated confidence intervals. We then compare our confidence estimators with other existing confidence and AD estimators on two well-studied biological benchmark datasets from MHC–peptide binding prediction and QSAR. Our results suggest that CONFINE and CONFIVE perform comparable to or better than previously proposed estimators, given a sufficient amount of training data. We also show that confidence intervals are a very intuitive and informative way to express the reliability of individual predictions. To illustrate the universal character of CONFINE and CONFIVE, we apply them to linear as well as non-linear regression. The results confirm that the confidence estimators presented here are able to estimate the reliability of predictions in terms of their error and thus can improve the user’s confidence in prediction methods in computational biology.

An open-source implementation of both methods is available in the R package confReg (http://cran.r-project.org/web/packages/confReg/index.html).

### Related Work

When the response of a novel instance 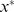 has been predicted using a trained regression model, confidence estimators try to determine the reliability of this particular prediction. A confidence estimator is a function 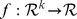, where the input is a test instance 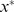 and the output is a confidence score 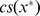. Note that confidence estimators and AD estimators do not try to predict the exact error of a prediction itself. Instead, they require predictions with a low error to have a small confidence score and predictions with a high error to have a large confidence score. Scores determined by different estimators are not necessarily comparable nor interpretable. Determining a threshold for the applicability domain of a model is, hence, often very vague. Instead of relying on non-interpretable scores that cannot be interpreted by a user, we propose an approach of translating confidence scores into interpretable confidence intervals, a more intuitive expression of confidence. We will briefly discuss related work before introducing our novel concepts for confidence estimation in ‘Materials and Methods’.

A traditional approach to estimate ADs is based on the number of neighbors (NoNN) of 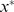 in the training dataset [4], [6]. It is based on the assumption that the prediction error is lower for instances within a more populated subspace and higher for instances within a sparsely populated subspace. The size of the subspace can either be given in advance or determined in a cross-validation.

An intuitive approach to confidence estimation calculates the absolute difference of the predicted response 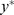 and the average response of the 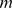 nearest neighbors [6]:
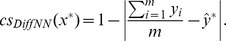



If the error difference is relatively low, the prediction is assumed to be reliable.

Another popular class of AD estimators are distance-based [7], [8]. One popular representative is the average Euclidean distance (AvgDist) to instances in the training dataset [4]:
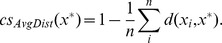



It is assumed that predictions of instances with a large average distance to the training dataset are more erroneous since the model has to extrapolate.

Bosni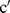 and Kononenko [9] introduced a method of confidence estimation based on the local sensitivity of a regression model. Predictions are rated as confident if the local variance (LocalVar) introduced by local changes in the learning data is considerably low. Local changes are introduced by adding the test instance to the training datasets using different response values. For each change, the model is re-trained and the original prediction is repeated. This obviously requires a lot of runtime resulting in a huge computational overhead. A detailed description of this approach can be found in the Supporting Information S1. Note, when this method is applied in combination with linear regression, it approximates the predictive variance of the regression model.

Later, the same authors proposed a confidence estimator that performs a leave-one-out cross-validation on the 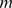 nearest neighbors of 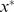 (LocalCV) [6]. It does not consider errors made by the overall model, but errors made by locally trained models. The local environment 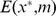 of 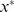 in training dataset 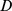 is defined as a set of the 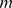 nearest neighbors, the 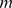 instances 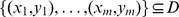 with the smallest Euclidean distance 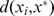 to 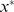. For every neighbor 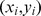 in the local environment 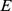, a regression model is trained on 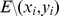. Then, the response 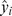 of 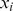 is predicted with this model and the absolute prediction error 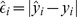 is calculated. By weighting the instances according to their distance to 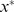, we receive the following confidence estimator:
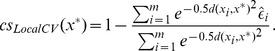



Obviously, estimation with LocalCV requires long runtimes, since the leave-one-out cross-validation has to be repeated for every single instances 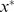. In our work, we set 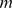 to 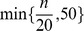 to reduce runtime.

Last but not least, the variance of multiple regression models combined by bootstrap aggregation, also known as bagging, has been used to estimate confidences [10], [11]. Given a training dataset 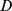, we create 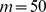 new datasets 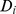 of the same size as 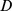 by uniformly sampling with replacement instances from 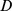. Every dataset 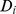 is used to train a regression model and to predict our novel instance 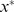, resulting in 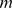 predicted response values 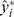. Since we expect agreement among the predictors in case of a reliable prediction, the final confidence estimator is based on the variance of the predicted responses:
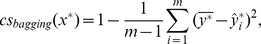
where 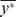 denotes the mean of all predictions 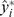.

The presented confidence estimation approaches show different advantages and disadvantages: Estimators NoNN, DiffNN, and AvgDist are obviously very fast. However, they do not consider the prediction model and, hence, might be less sensitive to model specific prediction behavior. Moreover, NoNN and AvgDist assume that a populated subspace leads to a better prediction quality, which might be wrong if the responses in the small subspace show a very large variance. Similar, a large variance of responses in the local neighborhood can lead to false estimates by DiffNN. Confidence estimators LocalVar, LocalCV, and bagging are more involved since they consider the used prediction model. As a consequence, they require far more runtime. These three estimators analyze the variance of the prediction model using different approaches. However, none of these approaches take actual prediction errors into account.

Note that several other estimation methods, which are mostly modified versions of the above estimators, have been introduced in the past. A more comprehensive overview of these methods is given in the Supporting Information S1.

## Materials and Methods

In the following, we introduce our two novel confidence estimators, CONFINE and CONFIVE, and how their output is transformed into confidence intervals. Since both estimators are model-independent, they require some regression model to make predictions. In the first part of this work, we apply all confidence estimators together with linear least square regression. Let 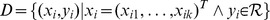 denote a given training dataset, with 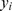 being the response values and 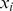 the input features. First, we select an appropriate feature set by minimizing the mean squared error (MSE) in a cross-validation (see Supporting Information S1 for details). Then, a linear regression model is trained via ordinary least squares on the resulting training dataset. The performance of the trained model is subsequently accessed by predicting the response values of a test dataset, which is disjoint from 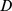. Later in the manuscript, we also apply our confidence estimators to non-linear support vector regression (SVR) model with a Gaussian radial basis kernel.

### Errors of Nearest Neighbors

Our first confidence estimator is called CONFINE (CONFidence estimation based on the Neighbors’ Errors). It is based on the MSE in the local environment of 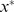 in the training dataset. We simply analyze how well the model fits the surrounding data and transfer this error to our test instance 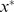. It has been adapted from Dimitrov et al. [5], who proposed a similar approach for classification. If the MSE of the 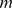 nearest neighbors is already very high, we do not expect the model to be very good on novel instances either. Thus, a large error in the local environment results in a low confidence score, whereas a low error results in a large score:
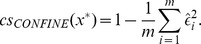



The prediction errors 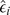 can be obtained by predicting the response values of the training dataset using a model trained on the same data or by performing a cross-validation on the training data. The optimal value of 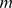 is obtained using five two-fold cross-validations on the training dataset by averaging the values of 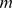 resulting in the highest estimation quality of each fold. We chose to use two-fold cross-validations to have a large test set in the optimization process.

We believe that CONFINE is very powerful since it considers actual errors made by the model instead of analyzing only the variance of predictions. If 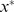 lies within a populated subspace, CONFINE is able to interpolate the error based on very similar instances. On the other hand, if 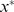 lies within a sparsely populated subspace, we transfer the errors of instances within these sparsely populates subspace, which we are likely to show larger absolute errors.

Note that we also propose a modified version of this estimator, which uses a kernel density estimate. Instead of relying on a fixed local environment, we weight instances according to their distance to 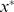, such that we put more weight on instances that are close to 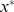 (see Supporting Information S1 for details).

### Variance of in the Environment

Our second confidence estimator is called CONFIVE (CONFIdcence estimation based on the Variance in the Environment). It is based on the variance of the response values of the 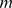 nearest neighbors of 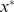. CONFIVE assumes that a large variance of the responses in a local region is difficult to model with a regression approach. This is especially true if a linear model is applied. Thus, large variances result in a low confidence score, whereas small variances result in a large score:
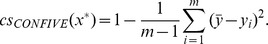



The optimal value of 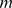 is also obtained using five two-fold cross-validations. As an alternative, we propose a version of CONFIVE based on a kernel density estimate (see Supporting Information S1).

### Confidence Intervals

When the response of a novel instance 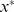 has been predicted using the trained regression model, we apply our confidence estimators for this particular prediction. Since obtained confidence scores 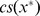 determined by different estimators are not necessarily comparable nor interpretable, we calculate normalized confidence scores 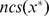 as described below. We first predict the responses of the training data and then apply the confidence estimator for each prediction. The normalized confidence score 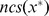 of a novel instance 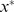 is then calculated by determining the fraction of predictions from the training dataset with a smaller confidence value than 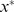. Thus, an 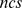 of 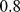 implies that 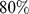 of the instances in the training dataset have been predicted with a smaller confidence value. Using this approach, we obtain meaningful and interpretable scores which lie between zero and one.

Normalized confidence scores are useful indicators of the prediction error. We assume that the higher the score of a predicted instance, the more likely this instance was predicted with a small error. Still, it is not obvious how such a score relates to an actual error. For example, given an 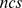 of 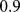, it is not obvious how large the actual prediction error is.

Confidence intervals are a much more intuitive concept than arbitrary scores. Instead of predicting only the response 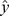 and the corresponding normalized confidence score 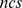, we predict an interval based on 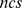 which includes the correct response value 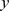 with a probability of 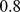. Since reliable predictions with a large 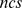 have, on average, a smaller squared error, we expect them to have smaller confidence intervals. We can relate an 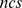 to confidence intervals (e.g., 80% confidence intervals) as follows.

Since we assume that instances with a similar confidence score have a similar error, we estimate confidence intervals based on the errors of predictions with similar confidence score. In a first step, we predict the responses of the training instances using a model trained on the training dataset. Subsequently, the normalized confidence scores of all training instances are first estimated using a confidence estimator based on the training data and then sorted in ascending order 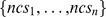. For every possible normalized confidence score 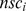, we collect the errors of instances with an 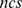 of 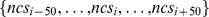 where possible. Otherwise, we use a reduced set of errors. Based on this set of errors 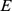, we calculate the 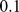 quantile 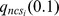 and the 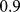 quantile 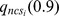 as interval borders. By using empirical quantiles, we do not assume a normal distribution and, hence, are independent of the underlying error distribution.

When predicting the response 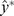 and the confidence score 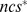 of a novel instance 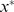, we calculate the 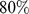 confidence interval as 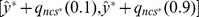.

Note, in case of CONFINE, we could also simply utilize only the errors of the nearest neighbors of an instance to estimate intervals. However, we found this naive estimate to perform worse than the above described approach, possibly due to the smaller set of acquired errors.

### Evaluation

The quality of the predicted confidence intervals is measured based on a simple requirement: the more erroneous a prediction is, the larger should be the confidence interval. Hence, predictions with a large squared error should yield a broad confidence interval, while predictions with a low squared error are assumed to have a small confidence interval. Since, to the best of our knowledge, there exists no appropriate evaluation metric, we measure the quality of confidence interval estimation as follows.

An intuitive measure of this requirement is the Pearson product-moment correlation coefficient 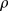. Consequently, we can assess the quality of estimates by calculating the correlation 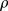 between the absolute prediction errors 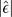 and the widths of the corresponding confidence intervals 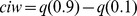. The resulting correlation is then normalized by the correlation obtained by a perfect confidence estimator. To simulate a perfect confidence estimator, we re-order the prediction errors and confidence interval widths in a way that 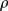 is maximized. This can be simply done by sorting 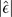 and 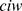. We define the *confidence–error correlation* (CEC) as
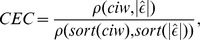
where *sort* is an arbitrary sorting function. Since we wish to calculate an 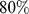 confidence interval, we obviously also require about 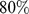 of the test errors to lie within the confidence interval.

**Figure 1:**
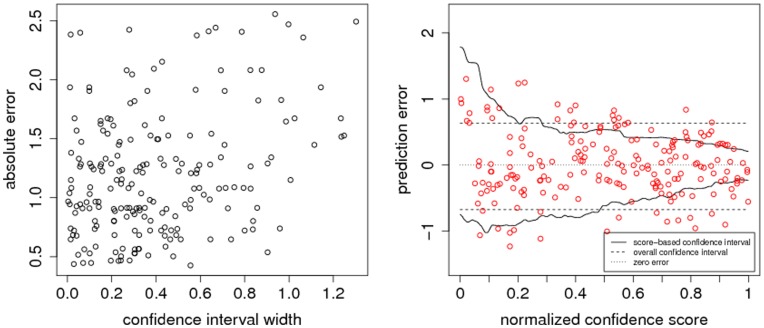
Example of estimating confidence intervals. In this example, we estimated the confidence intervals of 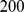 instances. The left-hand plot shows the confidence interval widths and the corresponding absolute errors. The corresponding CEC equals 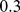. Although the CEC is not very large, it is possible to see an increased number of small confidence intervals for predictions with a low error. In the right-hand plot, the estimated confidence interval borders are displayed. In addition, every prediction defined by its prediction error and its normalized confidence score is depicted by a red circle. On average, the absolute error is smaller for predictions with a high 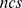 and a small confidence interval.

In Fig. 1, we show absolute errors as a function of confidence interval widths estimated by CONFINE. At a first glance, the resulting CEC of 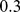 does not seem all that impressive. It should be noted, however, that we do not expect a perfect correlation between the error and the confidence interval width. It is only required that the error is smaller than the confidence interval. While correlation is thus obviously not the perfect measure, we used it because of its rather intuitive nature.

The CEC should not be confounded with the prediction quality of a model itself. Even if a model performs almost perfect, it is not necessarily easier to estimate its prediction errors. It should also be noted that already a CEC of 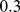 can lead to a considerably reduced confidence interval for confident predictions, as can also be seen in the left-hand plot of Fig. 1.

In many real world applications, users are only interested in highly reliable predictions. To account for that, we also measure the *confidence-associated prediction improvement* (CAPI). Therefore, we calculate by what percentage the MSE is reduced if we consider only the top 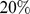 predictions, i.e. the 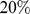 predictions with the smallest confidence intervals.

### Datasets

We benchmarked our methods on three different types of datasets: a synthetic dataset, several QSAR datasets, and a dataset stemming from immunoinformatics (MHC–peptide binding). The synthetic dataset was created using the Friedman function [12] with different levels of Gaussian noise:
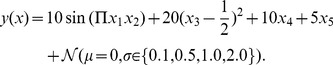
(1)


This test function has five relevant features 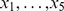, where two are linear and three are non-linear. We created datasets of different sizes 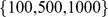 by sampling 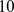, 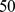, 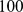, or 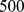 features from 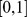 uniformly. The response values were calculated by applying the Friedman function to the first five features, additional features 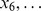 have no influence on the response value. Since we are able to scale properties such as the size, the number features, and the noise, this dataset is well suited to measure the influence of these attributes.

Furthermore, we used eight popular benchmark datasets from QSAR [13], which consist of 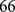 to 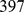 chemical compounds and corresponding experimentally obtained response values. We calculated up to 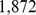 features using DragonX 1.4.0 [14].

Our third type of data is MHC–peptide binding data. We extracted peptides of length nine with experimentally verified binding affinities to molecules from 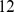 different MHC class I alleles from the IEDB benchmark dataset [15]. We chose the 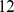 HLA alleles for which more than 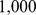 examples are available: HLA-A*01∶01, HLA-A*02∶01, HLA-A*02∶02, HLA-A*02∶03, HLA-A*02∶06, HLA-A*03∶01, HLA-A*11∶01, HLA-A*31∶01, HLA-A*33∶01, HLA-A*68∶01, HLA-A*68∶02, and HLA-B*07∶02. Each position in the peptide sequence is encoded by a vector consisting of 19 zeros and a one corresponding to the amino acid at this position and, further, six values encoding for the hydrophobicity and charge of the amino acid. The resulting datasets contain 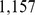 to 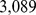 instances, each encoded by 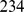 features.

We chose datasets from QSAR and MHC-I binding prediction since they have quite different properties with respect to the number of data points, the size of the input feature space, and the coverage of that input space. Peptides are defined by their sequence, which is usually encoded by a simple 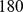-dimensional binary feature vector. In contrast, chemical compounds are more complex, since their 3D structure has to be encoded into features, leading to more than 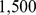 feature values for each instance. Moreover, the feature values of compounds can take values that might be unique within the whole dataset. In contrast, it is very unlikely that there is no peptide with the same amino acid at one particular position in a dataset of more than 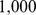 peptides. Further, the amount of training data used for QSAR models is usually very small, often only 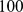 to 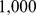 instances, while MHC binding data might be given for more than 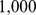 peptides. See also Table 1 in the Supporting Information S1 for detailed dataset sizes.

**Table 1:** Performance of confidence estimators on artificial data with different properties.

	*n*≤100	*n*>100	*m*≤10	*m*>10	σ<1.0	σ≥1.0	best
CONFINE	0.05	0.22	0.19	0.05	0.21	0.15	0.30
CONFIVE	−0.02	0.05	0.03	−0.01	0.04	0.02	0.07
AvgDist	0.02	0.12	0.10	0.03	0.11	0.08	0.16
Bagging	0.11	0.20	0.18	0.11	0.19	0.16	0.25
Diff5NN	0.01	0.17	0.14	0.02	0.14	0.11	0.29
LocalCV	0.01	0.05	0.04	0.02	0.04	0.03	0.05
LocalVar	0.00	0.12	0.10	−0.00	0.09	0.08	0.16
NoNN	0.05	0.12	0.12	0.03	0.11	0.09	0.16

For every confidence estimator, we calculated the average CEC by considering datasets with a different number of instances 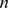, a different number of selected features 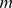, and a different noise level 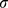. In the last column, we show the average CEC for the best parameter combination (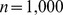, 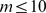, 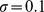).

## Results and Discussion

### Influence of Dataset Size, Features, and Noise

In an initial experiment, we analyzed how the introduced confidence estimators are influenced by the dataset size, the number of features, and noise in the data. The experiment was performed on the synthetic dataset, which gives us full control over these parameters. We performed five nested five-fold cross-validations on randomly generated artificial datasets, each with a different number of instances, features, and noise levels in the response variable, resulting in 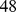 combinations. The estimation quality of the confidence estimators in terms of the average CEC (avgCEC) are shown for different parameter combinations in Table 1. Details on qualities regarding the confidence associated prediction improvement (CAPI) can be found in the Supporting Information S1.

We found that the dataset size has the strongest influence on the estimation performance. On very small datasets with only 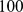 instances, the estimators yield an avgCEC of 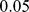. When considering datasets with more than 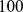 instances, the avgCEC of all estimators increases to 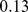. In addition, we observed a CAPI of 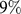 on small datasets and a CAPI of 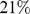 if more than 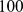 training instances are given. Still, not all estimators are equally sensitive to the dataset size. While the avgCEC of estimator CONFIVE is only slightly influenced by the dataset size, the avgCEC of CONFINE increases by 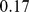 when considering sufficiently large datasets. For large datasets, CONFINE shows a CAPI of 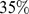. Moreover, note that when the dataset size is increased from 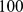 to 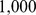 instances, the standard deviation of the CECs decreased from 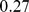 to 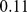.

We also observed that noisy features and noise in the responses have an influence on the quality of confidence estimates. Particularly when the initial number of features was high or the dataset size was low, noisy, non-predictive features were included in the feature set. When more than 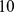 features were selected, the avgCEC of all estimators decreased by 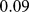. Similar results were obtained regarding the noise in the data. When random values with a low standard deviation (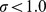) were added to the data, the avgCEC was up to 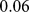 larger compared to avgCECs obtained on data with a higher noise level.

As expected, when we considered only datasets with 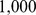 instances, 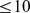 selected features, and a noise level of 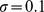, all estimators yield their best performance. In particular, CONFINE performs well, yielding an avgCEC of 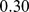 and a CAPI of 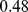, i.e. the 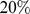 of predictions that had the smallest confidence intervals exhibited a 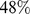 lower MSE than an average prediction.

From our results, we can conclude that – not surprisingly – a larger amount of training data results in more robust confidence estimates and higher confidence estimation quality. In addition, a good feature representation and a low noise level support confidence estimation. Clearly, these properties are not independent of each other. Distinguishing between informative and non-informative features is easier for large datasets, since the difference between noise and information becomes more evident. The same holds for datasets with a low level of noise, resulting in less noisy features. Since most confidence estimators discussed here inspect local properties of the input space, they rely on good feature representation. If noisy features are part of the feature set, instances in the local environment are not necessarily similar to the test instance and, thus, provide no reliable confidence information. Furthermore, given more instances in the dataset, we can define a local environment with a smaller diameter since the density of instances is higher. Consequently, the nearest neighbors are more similar to the test instance and contain more relevant confidence information.

### Evaluation on Biological Data

To compare CONFINE and CONFIVE with existing confidence estimators, we performed five nested five-fold cross-validations on the MHC datasets and the QSAR datasets.

Due to the different properties of the biological datasets, the results are rather diverse (see Table 2). On the MHC datasets, our estimators CONFINE and CONFIVE, as well as DiffNN, with an avgCEC of around 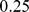, perform superior to all other estimators, which yield an avgCEC around 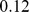. We summarized the improvement in CEC values across the MHC datasets with random effects models [16]. We found that CONFINE, CONFIVE, and DiffNN show a higher CEC of at least 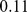, 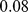, and 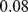, respectively, compared to all other methods. In all three cases the improvement was significantly greater than 0 with a p-value 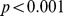. In addition, the best three confidence estimators show a CAPI of up to 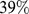, while the other estimators yield an average improvement of only 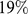. When summarizing the CAPI improvements across the datasets using random effect models, we found that CONFINE and CONFIVE perform significantly better than all other methods except of DiffNN with a p-value 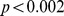. On the QSAR data, bagging performs best (p-value 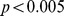), yielding an avgCEC of 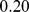, while estimators CONFINE, CONFIVE, and LocalCV perform second best, with avgCECs around 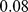 and p-values of 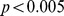, 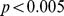, and 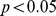, respectively. Since most estimators have been shown to be very sensitive to the dataset size, we also calculated the avgCEC considering only QSAR datasets with more than 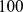 learning examples. On large QSAR datasets, the avgCEC of most estimators, except for bagging, is considerably improved. In the case of CONFINE and CONFIVE, the avgCEC improves to 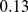 and 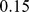, respectively. A similar trend can be observed when considering prediction improvement.

**Table 2:** Performance of confidence estimators on biological datasets.

Regression model	confidence	MHC			QSAR		
	estimator	CEC	CAPI	runtime [ms]	CEC	CAPI	runtime [ms]
LR	CONFINE	0.27	0.39	2	0.08	0.09	1
	CONFIVE	0.24	0.35	2	0.09	0.13	1
	AvgDist	0.11	0.18	2	−0.02	−0.10	1
	Bagging	0.13	0.18	1	0.20	0.35	1
	DiffNN	0.24	0.32	2	−0.00	−0.14	1
	LocalCV	0.16	0.27	214	0.08	0.10	353
	LocalVar	0.10	0.17	482	−0.08	−0.22	430
	NoNN	0.10	0.17	2	−0.03	−0.09	1
SVR	CONFINE	0.23	0.41	9	0.23	0.32	9
	CONFIVE	0.21	0.34	10	0.16	0.21	10
	AvgDist	0.12	0.23	9	0.02	0.03	12
	Bagging	0.21	0.50	374	0.15	0.17	3064
	DiffNN	0.24	0.35	9	0.10	0.20	10
	NoNN	0.22	0.18	9	0.12	0.14	44

For every confidence estimator, the avgCEC, the confidence associated prediction improvement (CAPI), and the time for an individual estimation in milliseconds on the MHC datasets and on the QSAR datasets is shown. For the upper part of the table, the estimators were applied together with linear regression (LR), whereas the number in the lower part were obtained using support vector regression with an RBF kernel (SVR).

Estimating confidences with CONFINE and CONFIVE is possible with only a minor computational overhead. Estimating the confidence intervals of one individual prediction requires about 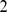 ms on a 2 GHz dual-core AMD Opteron with 4 GB of RAM using our R implementation. But also most other estimators need about 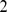 ms for an estimation. Only estimators LocalCV and LocalVar require more than 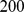 ms for an individual estimation. For each estimation, both estimators train multiple regression models, which results in a huge computational overhead. Note that bagging uses only predictions of multiple regression models and is faster than LocalCV and LocalVar as long as we rely on linear regression, as we will experience in the following section.

Our results suggest that CONFINE and CONFIVE often perform better than most other confidence estimators while being comparable in quality to bagging. Especially on the MHC datasets, where more than 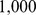 training examples are given, and on sufficiently large QSAR datasets, our methods yield high quality confidence estimates. In contrast, commonly used AD estimators such as AvgDist, DiffNN, and NoNN often fail to give reasonable error estimates. Interestingly, CONFIVE performs well on the biological datasets, while yielding a poor performance on artificial data. In addition, CONFINE and CONFINE require only a small computational overhead.

### Confidence Estimation for Non-linear Models

To show that CONFINE and CONFIVE can be also applied to non-linear regression models, we repeated the evaluation on the MHC and QSAR datasets using SVR. Since estimators LocalCV and LocalVar require too much runtime, we excluded them from this study. The parameters of the SVR and the estimators were optimized by performing nested cross-validations on the training dataset. Since optimizing SVRs requires more runtime, we restricted the evaluation to only one nested five-fold cross-validation. The results are shown in Table 2.

Similar to our previous results, confidence estimators CONFINE, CONFIVE, DiffNN, and bagging show the best overall performance. While the avgCEC of CONFINE and CONFIVE was comparable to our previous results on the MHC datasets, the avgCEC on the QSAR data was higher. In particular on the QSAR datasets, the avgCEC and CAPI of CONFINE is significantly larger than the avgCEC of all other methods except of DiffNN (p-value 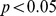), which shows large variance in its performance. Note that we again observed that CONFINE and CONFIVE performed better on larger QSAR datasets.

Confidence scores and confidence intervals could be predicted with only a small computational overhead using estimators CONFINE, CONFIVE, AvgDist, DiffNN, and NoNN. On the MHC and QSAR datasets they require between 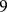 to 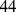 ms for an individual prediction. The different estimation times between estimators and the differences compared to our previous results using linear regression origin from the different number of features. Confidence estimation based on bagging requires the largest runtime of up to 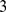 seconds for an individual prediction.

Our findings support the assumption that CONFINE and CONFIVE show similar behavior when being applied in combination with non-linear regression models. In particular, CONFINE shows again a very good and very robust performance, while being fast at the same time. Although confidence estimation based on bagging shows also a good performance, bagging is less practical for real world applications. If bagging is applied with a time-consuming regression model, runtimes can be considerably high. In contrast, CONFINE and CONFIVE perform independent of the actual regression model, making them even more interesting for real world application.

### Evaluation of Confidence Intervals

To show that a score-based 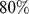 confidence interval contains as many instances as an interval estimated independently from a confidence score, we compared it with a general 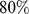 confidence interval. Therefore, we calculate the 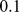 quantile and the 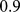 quantile of the squared errors of all training instances without considering the confidence scores. While the score-based confidence intervals are expected to be smaller for large 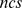, the general interval is always of the same size.

On the artificial dataset, we observed an almost equal fraction of 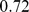 and 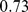 instances in the score-based interval and the general interval, respectively. If we consider only datasets with more than 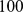 instances, we find about 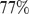 of the instances within both confidence intervals. Among the different confidence estimators, we could not find considerable differences. On the MHC datasets, a fraction of 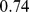 and 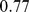 instances are covered by the score-based interval and general interval, respectively. In contrast, only 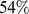 and 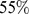 of the instances from the QSAR datasets fall into the respective confidence intervals. However, when considering only QSAR datasets with more than 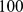 training examples, about 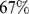 of the instances are within both confidence intervals.

Our results suggest that score-based confidence intervals contain the same fraction of instances as general confidence intervals. In particular, on large datasets, the fraction of instances within the confidence interval converges to 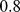. Further, since the widths of score-based confidence intervals are correlated with the absolute prediction error, they are a very intuitive measure of confidence.

### Predicting the Estimation Performance

Although confidence estimation can give valuable information in addition to plain response values, the quality of estimates differs from dataset to dataset. To answer the question whether we can predict the quality of confidence estimates, we compared the CECs obtained from the training data (CEC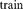) with the CECs obtained from the corresponding test data (CEC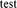) for all estimators.

On the artificial dataset, we observed an average correlation coefficient 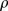 between CEC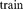 and CEC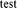 of 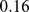. When considering only datasets with more than 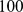 training examples, the average 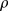 increased to 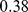. The same trend could be observed in the biological datasets. For the considerably large MHC datasets, we received an average 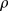 between CEC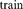 and CEC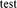 of 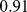, while no correlation appeared for the fairly small QSAR datasets. In particular, the training CECs of CONFINE and bagging show a comparably good correlation with their corresponding CECs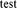 for all datasets. See Supporting Information S1 for more details.

If a sufficient amount of training data is available, the performance of confidence estimators is well correlated with their performance on the training data. This allows us to make an educated guess as to how a confidence estimator will behave on new data. In particular, the performance of CONFINE and bagging is quite predictable using performance information from the training data.

### Conclusion

Estimating the confidence in individual predictions is crucial for the interpretability of machine learning models. Confidence estimation has two main purposes: it yields reliable bounds on the error of *individual* predictions thus increasing the confidence of the user in predictions and it allows the selection of highly confident predictions. The latter can be very valuable if predictions for large datasets are made and confidence can serve as a selection criterion for experimental validation. For example, in the case of MHC binding prediction, a large number of high-affinity binders might be predicted and experimental validation might proceed based on the confidence in the prediction in order to confirm a larger number of good binders with fewer experiments.

In this work, we propose two novel confidence estimators for regression, CONFINE and CONFIVE. They determine normalized confidence scores and confidence intervals that help biologists to rate the reliability of an individual prediction. Both estimators are model-independent and can be applied with any regression model. In contrast to model-dependent confidence estimation methods, CONFINE and CONFIVE are computationally very efficient and can thus be added easily to existing predictors without a significant performance loss.

In an initial study on artificial data, we observed that CONFINE and CONFIVE, as well as other estimators, yielded a better estimation performance on large datasets. A sufficient amount of training data helps to identify irrelevant features and increases the prospect of having adequate neighbors in the training dataset. We then compared CONFINE and CONFIVE with other existing confidence and AD estimators on two benchmark MHC binding prediction and QSAR datasets. Our results suggest that CONFINE and CONFIVE give high quality confidence estimates if sufficient training data is available. Especially on the large MHC datasets, both estimators often perform better than existing methods. Similar results obtained using non-linear support vector regression demonstrate that CONFINE and CONFIVE can be applied to non-linear regression models as well. Only confidence estimation based on bagging performs comparably on the tested datasets. However, depending on the regression method used, bagging can require a huge computational overhead. We also have seen that confidence intervals estimated by our two methods are comparable to fixed confidence intervals, while having the advantage of giving a very intuitive measure of confidence.

Nevertheless, since properties differ from dataset to dataset, care needs to be taken when applying confidence estimators. Moreover, different needs might influence the choice of a confidence estimator. In cases where only outlier detection is required (i.e., where the prediction of an applicability domain is required), simple distance-based estimators might suffice. However, if enough data is given, one should exploit the advantages of having quantitative confidence estimates. Furthermore, since the quality of future confidence estimates by CONFINE and CONFIVE can be predicted if large training datasets are given, it can be checked in advance whether they yield satisfactory estimation quality for a given task.

It is still a long way towards highly accurate confidence estimators that work equally well on any kind of data. A combination of multiple confidence estimators as well as an automated selection [17] could improve both the quality and the robustness of the estimation. Further, predicting not only the size of errors but also their sign will increase the amount of information gained from a confidence estimator. As an alternative, signed error estimates can be used to correct the prediction results and might increase the prediction performance of the regression model.

Estimation of normalized confidence scores and confidence intervals is clearly a step forward, moving away from plain regression values and a discrete applicability information. In particular, confidence intervals provide a very intuitive representation of reliability, which can be easily interpreted by biologists. As a consequence, confidence information will help to increase the trust of biologists in *in silico* predictions. Distinguishing between confident and almost random predictions will also help biologists to choose suitable candidates for further experiments. We are convinced that confidence estimators will become standard for computational prediction models in the near future.

## Supporting Information

Supporting Information S1 (PDF)
