# Supplementary material for: No Longer Confidential: Estimating the Confidence of Individual Regression Predictions
Source: PLoS One. 2012 Nov 15;7(11):e48723. doi: 10.1371/journal.pone.0048723 (PMC3499506; doi:10.1371/journal.pone.0048723)
Supplement: Supporting Information S1 — (PDF) [file pone.0048723.s001.pdf]

Supporting Information to "No longer confidential: Estimating the Confidences of Individual Regression Predictions"

Sebastian Briesemeister[1,*], Jörg Rahnenführer[2], Oliver Kohlbacher[1]
[1] **Applied Bioinformatics Group, Center for Bioinformatics / Dept. of Computer Science, University of Tübingen, Germany**
[2] **Department of Statistics, TU Dortmund, Dortmund, Germany**
∗ **E-mail: briese@informatik.uni-tuebingen.de**

Materials and Methods

Linear regression

To obtain interpretable predictions, we use a linear regression model throughout this study. We are given a labeled dataset $D = \{(x_i, y_i) | x_i = (x_{i1}, \dots, x_{ik})^T \wedge y_i \in \mathbb{R}\}$, with $X = (x_1, \dots, x_n)^T$ being the design matrix and $Y = (y_1, \dots y_n)^T$ the vector of response variables. Linear regression assumes an approximately linear relationship between x_i and y_i such that:

$$y_i = \beta_0 + \beta_1 x_{i1} + \ldots + \beta_k x_{ik} + \epsilon_i = x_i^T \beta + \epsilon_i,$$

where β is the parameter vector and ϵ_i are the errors. The responses $\hat{y}$ of an instance x is then predicted by $x^T\beta$. The parameters β can be estimated via ordinary least squares $\hat{\beta} = (X^T X)^{-1} X^T y$. Ordinary least squares minimizes the sum of squared errors made by the model, defined by $\sum_i (y_i - \hat{y}_i)^2$. To avoid singularity, we use a very small Tikhonov regularizer of $\lambda = 10^{-10}$. Consequently, $\hat{\beta} = (X^T X + \lambda I)^{-1} X^T y$ is used for training the model.

Feature Selection

During the training of our linear model, we select features in a nested cross-validation. First, the quality of every feature is assessed by a three five-fold cross-validation using only this feature. The features are then sorted in ascending order of their average SE. Starting with the feature with the lowest SE, the features are iteratively added to the feature set. If the new feature set shows a higher average SE in three five-fold cross-validation than the previous feature set, the newly added feature is deleted. Since predictions are only interpretable in manual fashion if the feature number is relatively low, we set the maximum number of features to 50. The resulting feature set is used to train our linear model.

Confidence Estimators

Kernel-based Approaches of CONFIVE and CONFIVE

We also propose a modified version of both estimators based on a fixed kernel density estimate using a Gaussian kernel. Using the following approach, we put more weight on instances that are close to x^*:

$$\text{CONFIVE}^* = 1 - \frac{\sum_{i=1}^{n} e^{-0.5 d(x_i, x^*)^2} (\bar{y} - y_i)^2}{\sum_{i=1}^{n} e^{-0.5 d(x_i, x^*)^2}},$$

$$\text{CONFINE}^* = 1 - \frac{\sum_{i=1}^{n} e^{-0.5 d(x_i, x^*)^2} \hat{\epsilon}_i^2}{\sum_{i=1}^{n} e^{-0.5 d(x_i, x^*)^2}}.$$

To save runtime, we use both estimators with a fixed kernel-width. Using this approach might result in a slightly reduced estimation quality. However, both estimators require no optimization and are computationally very cheap.

Number of Nearest Neighbors

A traditional approach to estimate confidences utilizes the number of neighbors [1]. For this purpose, we define the local environment $E(x^*, d_E)$ as the set of instances from the training data with a maximum distance d_E to x^*. The optimal value for d_E can be found using the cross-validation scheme introduced in the methods section of the manuscript. The confidence value is calculated as follows:

$$\mathrm{NoN}(x) = |E|.$$

Instead of utilizing the number of neighbors (NoN), we can transform NoN into a density-based estimator using a Gaussian kernel [2]:

$$\mathrm{NoN}^* = \frac{1}{n}\sum_{i=1}^{n} e^{-0.5 d(x_i, x^*)^2}.$$

Distance-based Estimators

Distance-based estimators, which are often used for AD estimation, try to distinguish between outliers and instances within the domain. The mistrust in a prediction grows with its distance to the training data. The following two estimators express the distance to the training dataset as the minimum distance or average distance:

$$\mathrm{MinDist} = 1 - \min_i d(x_i, x^*) \text{ and}$$

$$\mathrm{AvgDist} = 1 - \frac{1}{n}\sum_{i}^{n} d(x_i, x^*).$$

A slightly more involved estimator puts a bias on closer instances:

$$\mathrm{AvgBiasedDist} = 1 - \frac{\sum_i^n e^{-3d(x_i, x^*)} d(x_i, x^*)}{\sum_i^n e^{-3d(x_i, x^*)}}.$$

Since the training dataset itself might already contain some outliers that are rather hard to predict, one can exclude such outliers from the confidence estimators. This is done by considering only instances that can be predicted with a prediction maximum error of $\hat{\epsilon}_m$ [3]. Let MinDistOF, AvgDistOF, and AvgBiasedDistOF denote these "outlier-free" versions of the previously introduced distance-based estimators. An optimal threshold for the prediction error $\hat{\epsilon}_m$ can be estimated using the previous introduced cross-validation scheme.

One-Class SVM

A generalization of the distance-based AD estimators based on a one-class SVM [4] was introduced by Fechner et al. [5], here denoted as 1-SVM. It utilizes the decision value of a one-class SVM with a Gaussian kernel trained on the training dataset. The parameters of the SVM and the Gaussian kernel can be optimized in a cross-validation. However, the optimization of all parameters via grid search comes with an increase in runtime.

Difference to Nearest Neighbor Prediction

We also tested a modified version of DiffNN where m is set to five, called Diff5NN in the following.

Sensitivity Analysis of Local Variance and Local Bias

Bosnić and Kononenko [6] introduced confidence estimation based on the local sensitivity of a regression model. Sensitivity analysis determines how much the model is affected if we modify the training dataset. By introducing a local change into the learning data, we can explore the sensitivity of the regression model in this very local area of the data. For this, we extend the training data by the predicted instance x^*. The response value of our new learning example is set to $\hat{y}^* + \delta(y_{max} - y_{min})$, where $\hat{y}^*$ is the predicted response value of x^*, δ is a sensitivity parameter, and y_{max} and y_{min} are the maximum and minimum response values of the training data, respectively. To measure the effects of this change, we predict the response value $\hat{y}^*_\delta$ of x^* using a regression model trained on the updated dataset. This approach is not strictly model-dependent since it does not rely on certain properties of a model but treats the model as a black box.

For predicting confidence values, several updated datasets with different sensitivity parameters $\delta \in \Delta = \{0.01, 0.1, 0.5, 1.0, 2.0\}$ are used. The local variance approach estimates how strong the predicted response value is changed by local changes in the training data:

$$\text{LocalVar} = 1 - \frac{1}{|\Delta|} \sum_{\delta \in \Delta} (\hat{y}^*_\delta - \hat{y}^*_{-\delta}).$$

The bias estimator measures how unstable the prediction is by expressing the amount of local bias:

$$\text{LocalBias} = 1 - \left| \frac{1}{2|\Delta|} \sum_{\delta \in \Delta} (\hat{y}^*_\delta - \hat{y}^*) + (\hat{y}^*_{-\delta} - \hat{y}^*) \right|.$$

We use the absolute value of the local bias since we are not interested in which direction the predictor is more unstable.

Local Cross-Validation

Local regression models, such as locally weighted regression, are often able to increase the prediction accuracy by adapting to local properties of the input space [7]. This idea has been adapted for a confidence estimator [2]. Similar to CONFINE, it calculates the error of the m nearest neighbors. However, it does not consider errors made by a model trained on all data, but uses errors made by a locally trained model. Thus, it tests whether a local part of the input space can in general be modeled with the given regression model. The errors of the local model are calculated by a leave-one-out cross-validation. For every neighbor $(x_i, y_i) \in E$, a regression model is trained on $E \setminus (x_i, y_i)$. Then, the response $\hat{y}_i$ of x_i is predicted with this model and the absolute prediction error $\hat{\epsilon}_i = |\hat{y}_i - y_i|$ is calculated. By weighting the instances according to their distance to x^*, we receive the following confidence estimator:

$$\text{LocalCV} = 1 - \frac{\sum_{i=1}^{m} e^{-0.5 d(x_i, x^*)^2} \hat{\epsilon}_i}{\sum_{i=1}^{m} e^{-0.5 d(x_i, x^*)^2}}.$$

Obviously, estimation with LocalCV requires long runtimes, since the leave-one-out cross-validation has to be repeated for every single instances x^*. To reduce the runtime, we set m to $\min\{\frac{n}{20}, 50\}$.

Predictive Variance

A classic approach of confidence estimation is the use of the predictive variance of Bayesian models. In particular, Gaussian processes have been successfully applied for AD estimation [8]. However, in contrast to the presented confidence estimators above, this way of estimation is strictly model-based and can only be applied if it is possible to estimate the models predictive variance. If the predictive variance is very small, we would assume the model to be very confident about this prediction. In contrast, if the variance

is rather high, the confidence is low. For a linear regression model, the predictive variance is defined as $x^*(X^TX)^{-1}x^{*T}$, where X is the design matrix of our linear regression.

We did not consider the predictive variance in this work for two reasons: First, confidence estimation using the predictive variance is only possible for certain models and, hence, not independent of the model as the other presented estimators. Second, although the confidence values estimated by LocalVar do not equal the predictive variances of the linear regression, it is safe to assume that LocalVar approximates the behavior of the predictive variance. In our experiments, we observed that LocalVar and the predictive variance represented the same order of the instances, regarding their error. Thus, both estimation approaches resulted in the same confidence scores and, consequently, the same CEC in an evaluation.

Table 1. Sizes of Biological Datasets

data type	name	size
QSAR	ACE	114
QSAR	ACHE	111
QSAR	BZR	163
QSAR	COX2	322
QSAR	DHFR	397
QSAR	GPB	66
QSAR	THERM	76
QSAR	THR	88
MHC	HLA-A*01:01	1157
MHC	HLA-A*02:01	3089
MHC	HLA-A*02:02	1447
MHC	HLA-A*02:03	1443
MHC	HLA-A*02:06	1437
MHC	HLA-A*03:01	2094
MHC	HLA-A*11:01	1985
MHC	HLA-A*31:01	1869
MHC	HLA-A*33:01	1140
MHC	HLA-A*68:01	1141
MHC	HLA-A*68:02	1434
MHC	HLA-B*07:02	1262

This table shows the sizes of the QSAR and MHC datasets. Note that all datasets have one real numbered response variable.

Results

The performance of all estimators from the manuscript and the supplementary material on the artificial dataset is shown in Table 2.

The results of all estimators on the MHC-I binding data and the QSAR datasets regarding the CEC and CAPI are shown in Tables 4 and 5.

See Table 6 and 7 for the correlations of the test CECs with the training CECs using linear regression and support vector regression, respectively.

Table 2. CEC of confidence estimators on artificial data with different properties

	$n \leq 100$	$n > 100$	$m \leq 10$	$m > 10$	$\sigma < 1.0$	$\sigma \geq 1.0$	best
CONFINE	0.05	0.22	0.19	0.05	0.21	0.15	0.30
CONFINE*	0.07	0.23	0.20	0.06	0.21	0.16	0.28
CONFIVE	-0.02	0.05	0.03	-0.01	0.04	0.02	0.07
CONFIVE*	-0.03	0.01	-0.00	-0.02	0.01	-0.01	0.02
1-SVM	0.00	0.12	0.11	-0.02	0.10	0.08	0.17
AvgBiasedDist	0.01	0.03	0.03	0.01	0.04	0.02	0.05
AvgBiasedDistOF	0.02	0.08	0.07	0.01	0.09	0.05	0.13
AvgDist	0.02	0.12	0.10	0.03	0.11	0.08	0.16
AvgDistOF	0.05	0.11	0.11	0.03	0.11	0.09	0.14
Bagging	0.11	0.20	0.18	0.11	0.19	0.16	0.25
Diff5NN	0.01	0.17	0.14	0.02	0.14	0.11	0.29
DiffNN	-0.01	0.14	0.12	-0.04	0.11	0.08	0.27
LocalBias	0.01	0.02	0.01	0.02	0.03	0.01	0.03
LocalCV	0.01	0.05	0.04	0.02	0.04	0.03	0.05
LocalVar	0.00	0.12	0.10	-0.00	0.09	0.08	0.16
MinDist	-0.01	0.04	0.03	0.00	0.03	0.02	0.04
MinDistOF	0.01	0.08	0.07	0.01	0.09	0.04	0.13
NoNN*	0.01	0.12	0.11	-0.00	0.12	0.07	0.16
NoNN	0.05	0.12	0.12	0.03	0.11	0.09	0.16
PredVar	0.00	0.12	0.10	-0.00	0.09	0.08	0.16

For every confidence estimator, we calculated the average CEC by considering datasets with a different number of instances n, a different number of selected features m, and a different noise level σ. In the last column, we show the average CEC for the best parameter combination ($n = 1,000$, $m \leq 10$, $\sigma = 0.1$).

References

1. Sheridan R, Feuston B, Maiorov V, Kearsley S (2004) Similarity to molecules in the training set is a good discriminator for prediction accuracy in QSAR. J Chem Inf Comput Sci 44: 1912–1928.

2. Bosnić Z, Kononenko I (2008) Comparison of approaches for estimating reliability of individual regression predictions. Data Knowl Eng 67: 504–516.

3. Dragos H, Gilles M, Alexandre V (2009) Predicting the predictability: a unified approach to the applicability domain problem of QSAR models. J Chem Inf Model 49: 1762–1776.

4. Schölkopf B, Smola A, Williamson R, Bartlett P (2000) New support vector algorithms. Neural Comput 12: 1207–1245.

5. Fechner N, Jahn A, Hinselmann G, Zell A (2010) Estimation of the applicability domain of kernel-based machine learning models for virtual screening. J Cheminform 2: 2.

6. Bosnić Z, Kononenko I (2008) Estimation of individual prediction reliability using the local sensitivity analysis. Appl Intell 29: 187–203.

7. Atkeson C, Moore A, Schaal S (1997) Locally weighted learning. Artif Intell Rev 11: 11–73.

8. Schwaighofer A, Schroeter T, Mika S, Laub J, Ter Laak A, et al. (2007) Accurate solubility prediction with error bars for electrolytes: A machine learning approach. J Chem Inf Model 47: 407–424.

Table 3. Confidence associated prediction improvement of confidence estimators on artificial data with different properties

	$n \leq 100$	$n > 100$	$m \leq 10$	$m > 10$	$\sigma < 1.0$	$\sigma \geq 1.0$	best
CONFINE	0.11	0.35	0.31	0.14	0.37	0.24	0.48
CONFINE*	0.13	0.38	0.32	0.17	0.37	0.27	0.48
CONFIVE	0.02	0.09	0.08	0.02	0.12	0.05	0.12
CONFIVE*	0.03	0.01	0.03	-0.02	0.08	-0.00	0.04
1-SVM	0.08	0.22	0.21	0.02	0.21	0.16	0.25
AvgBiasedDist	0.13	0.06	0.09	0.06	0.11	0.07	0.07
AvgBiasedDistOF	0.07	0.16	0.14	0.06	0.21	0.10	0.21
AvgDist	0.07	0.22	0.19	0.08	0.21	0.16	0.25
AvgDistOF	0.12	0.22	0.21	0.07	0.22	0.17	0.25
Bagging	0.23	0.26	0.25	0.23	0.32	0.23	0.29
Diff5NN	0.04	0.22	0.18	0.06	0.20	0.15	0.35
DiffNN	0.04	0.20	0.18	-0.01	0.17	0.14	0.34
LocalBias	0.04	0.05	0.06	-0.01	0.09	0.03	0.05
LocalCV	0.06	0.12	0.11	0.07	0.12	0.10	0.11
LocalVar	0.06	0.21	0.19	0.01	0.17	0.15	0.25
MinDist	0.11	0.08	0.09	0.08	0.09	0.09	0.05
MinDistOF	0.05	0.15	0.13	0.04	0.21	0.08	0.23
NoNN*	0.10	0.22	0.21	0.06	0.21	0.17	0.24
NoNN	0.16	0.22	0.22	0.10	0.24	0.18	0.26
PredVar	0.06	0.21	0.19	0.01	0.17	0.15	0.25

For every confidence estimator, we calculated the confidence associated prediction improvement (CAPI) by considering datasets with a different number of instances n, a different number of selected features m, and a different noise level σ. In the last column, we show the average CAPI for the best parameter combination ($n = 1,000$, $m \leq 10$, $\sigma = 0.1$).

Table 4. Performance of confidence estimators on biological datasets using linear regression

confidence	MHC			QSAR		
estimator	CEC	CAPI	runtime [ms]	CEC	CAPI	runtime [ms]
CONFINE	0.27	0.39	2	0.08	0.09	1
CONFINE*	0.23	0.33	2	0.07	-0.08	1
CONFIVE	0.24	0.35	2	0.09	0.13	1
CONFIVE*	0.17	0.22	2	0.08	0.11	1
1-SVM	0.02	-0.02	1	-0.08	-0.23	1
AvgBiasedDist	0.03	0.00	3	-0.04	-0.17	1
AvgBiasedDistOF	0.00	0.01	3	0.02	0.00	1
AvgDist	0.11	0.18	2	-0.02	-0.10	1
AvgDistOF	0.02	0.00	2	-0.03	-0.02	1
Bagging	0.13	0.18	1	0.20	0.35	1
Diff5NN	0.16	0.17	2	0.05	0.13	1
DiffNN	0.24	0.32	2	-0.00	-0.14	1
LocalBias	0.08	0.11	481	0.01	-0.05	429
LocalCV	0.16	0.27	214	0.08	0.10	353
LocalVar	0.10	0.17	482	-0.08	-0.22	430
MinDist	0.04	0.05	2	-0.04	-0.24	1
MinDistOF	0.01	0.02	2	0.03	0.05	1
NoNN*	0.07	0.09	2	-0.02	-0.06	1
NoNN	0.10	0.17	2	-0.03	-0.09	1
PredVar	0.10	0.17	1	-0.08	-0.22	1

For every confidence estimator, the avgCEC, the confidence associated prediction improvement (CAPI), and the time for an individual estimation in miliseconds on the MHC datasets and on the QSAR datasets is shown.

Table 5. Performance of confidence estimators on biological datasets using support vector regression

confidence	MHC			QSAR		
estimator	CEC	CAPI	runtime [ms]	CEC	CAPI	runtime [ms]
CONFINE	0.23	0.41	9	0.23	0.32	9
CONFINE*	0.11	0.18	9	0.15	0.09	9
CONFIVE	0.21	0.34	10	0.16	0.21	10
CONFIVE*	0.08	0.28	9	0.11	0.07	10
AvgDist	0.12	0.23	9	0.02	0.03	12
Bagging	0.21	0.50	374	0.15	0.17	364
DiffNN	0.24	0.35	9	0.10	0.20	10
NoNN	0.22	0.18	9	0.12	0.14	44

For every confidence estimator, the avgCEC, the confidence associated prediction improvement (CAPI), and the time for an individual estimation in miliseconds on the MHC datasets and on the QSAR datasets is shown.

Table 6. Correlation of estimated performance and real performance using linear regression

	artificial data	artificial data $n > 100$	MHC data	QSAR data
CONFINE	0.13	0.43	0.88	0.06
CONFINE*	-0.38	-0.35	-0.53	0.15
CONFIVE	0.06	0.25	0.96	0.32
CONFIVE*	0.01	-0.05	0.92	0.12
1-SVM	0.21	0.33	0.52	-0.02
AvgBiasedDist	0.02	0.17	0.56	-0.15
AvgBiasedDistOF	0.19	0.25	-0.05	0.02
AvgDist	0.27	0.36	0.90	-0.21
AvgDistOF	0.31	0.42	0.10	0.17
Bagging	0.03	0.39	0.95	0.13
Diff5NN	0.46	0.65	0.90	0.12
DiffNN	0.51	0.74	0.95	-0.12
LocalBias	-0.02	0.08	0.88	0.17
LocalCV	0.04	0.13	0.90	0.10
LocalVar	0.24	0.35	0.88	-0.04
NoNN*	0.26	0.37	0.79	-0.15
NoNN	0.00	0.41	0.89	-0.12
PredVar	0.24	0.35	0.88	-0.04

For every confidence estimator, we calculated the correlation ρ between the CEC on test data (CEC_{test}) and the CEC on the training data using an estimator trained on the same data (CEC_{train}). In case of MinDist, CEC_{test} is obtained by averaging the CECs of a cross-validation on the training data.

Table 7. Correlation of estimated performance and real performance using support vector regression

	artificial data	artificial data $n > 100$	MHC data	QSAR data
CONFINE	0.22	0.44	0.84	0.12
CONFINE*	-0.33	-0.59	-0.56	0.04
CONFIVE	-0.11	0.21	0.94	0.39
CONFIVE*	-0.13	-0.30	0.60	-0.17
AvgDist	0.44	0.74	0.80	-0.10
Bagging	-0.33	-0.54	-0.47	0.05
DiffNN	0.03	0.27	0.90	0.17
NoNN	0.49	0.59	0.32	0.34

For the given confidence estimator, we calculated the correlation ρ between the CEC on test data (CEC_{test}) and the CEC on the training data using an estimator trained on the same data (CEC_{train}).
